# Supplementary material for: Wood-inspired metamaterial catalyst for robust and high-throughput water purification
Source: Nat Commun. 2024 Mar 6;15:2046. doi: 10.1038/s41467-024-46337-1 (PMC10917756; doi:10.1038/s41467-024-46337-1)
Supplement: Supplementary file 1 — Supplementary Information [file 41467_2024_46337_MOESM1_ESM.pdf]

Supplementary Information——

Wood-inspired metamaterial catalyst for robust and high-throughput water purification

Lei Zhang[1,2,3,4,†], Hanwen Liu[1,†], Bo Song[1,*], Jialun Gu[2,3,4], Lanxi Li[5], Wenhui Shi[1], Gan Li[2,3,4,6], Shiyu Zhong[2,3,4], Hui Liu[2,3,4], Xiaobo Wang[1], Junxiang Fan[1], Zhi Zhang[1], Pengfei Wang[7], Yonggang Yao[1, *], Yusheng Shi[1], Jian Lu[2,3,4,*]

[1]State Key Laboratory of Material Processing and Die & Mould Technology, School of Materials Science and Engineering, Huazhong University of Science and Technology, Wuhan, 430074, China

[2]CityU-Shenzhen Futian Research Institute, Shenzhen 518045, China

[3]Centre for Advanced Structural Materials, City University of Hong Kong Shenzhen Research Institute, Greater Bay Joint Division, Shenyang National Laboratory for Materials Science, Shenzhen 518057, China

[4]Department of Mechanical Engineering, City University of Hong Kong, Tat Chee Avenue, Kowloon, Hong Kong, China

[5]Department of Materials Science and Engineering, City University of Hong Kong, Tat Chee Avenue, Kowloon, Hong Kong, China

[6]Shenzhen Key Laboratory for Additive Manufacturing of High-performance Materials, Department of Mechanical and Energy Engineering, Southern University of Science and Technology, Shenzhen 518055, China

[7]Advanced Materials and Energy Center, China Academy of Aerospace Science and Innovation, Beijing 100176, China

[†]Lei Zhang and Hanwen Liu are contributed equally to this work.

Corresponding author's E-mail addresses: bosong@hust.edu.cn (Bo Song)
Corresponding author's E-mail addresses: yaoyg@hust.edu.cn (Yonggang Yao)
Corresponding author's E-mail addresses: jianlu@cityu.edu.hk (Jian Lu)

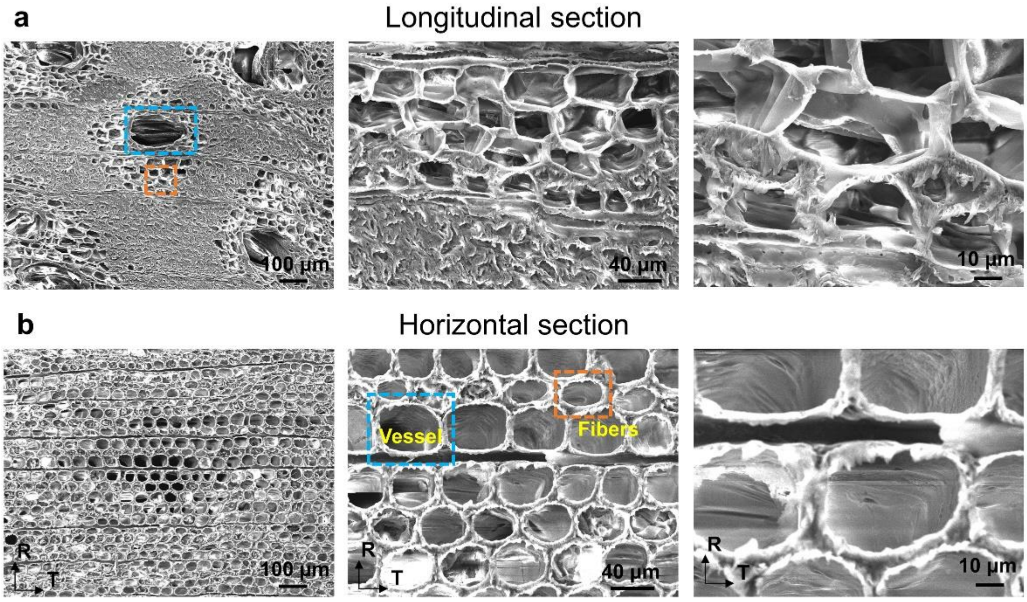

Supplementary Figure 1. **Scanning electron microscopy morphology of Douglas fir.** **a,** longitudinal section. **b,** horizontal section.

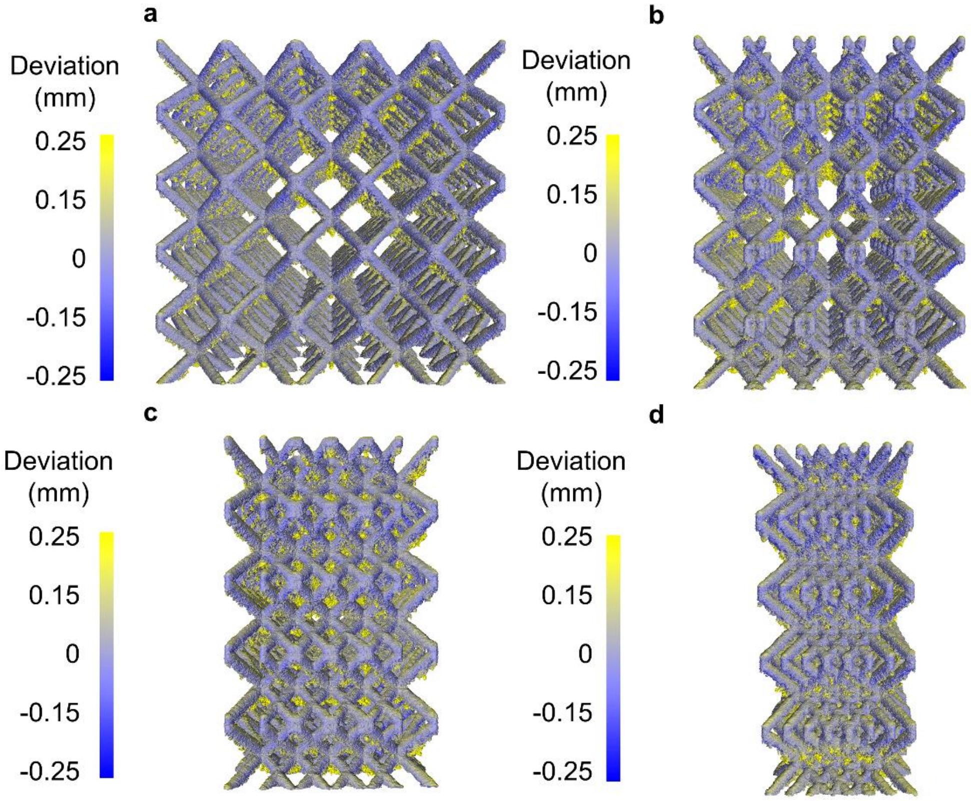

Supplementary Figure 2. **Comparative surface deviation nephogram of computer-aided design (CAD) and computed tomography (CT)-reconstructed 3D printed microlattice metamaterial models with different overlap rates. a,** 0% overlap rate. **b,** 30% overlap rate. **c,** 50% overlap rate. **d,** 70% overlap rate.

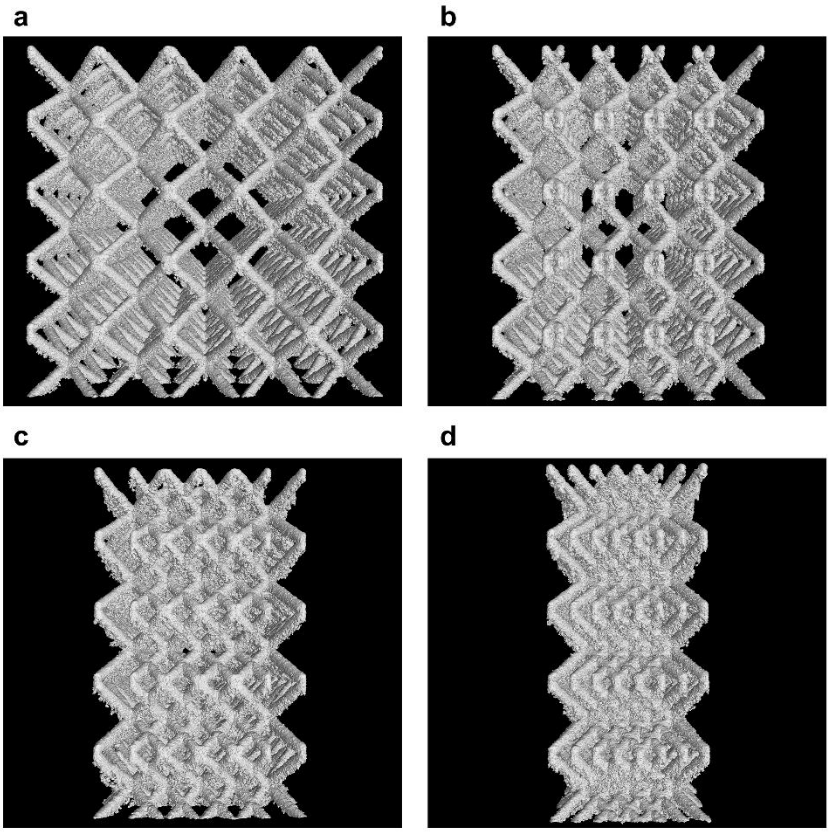

Supplementary Figure 3. Computed tomography (CT)-reconstructed 3D printed microlattice metamaterial models with different overlap rates. a, 0% overlap rate. **b,** 30% overlap rate. **c,** 50% overlap rate. **d,** 70% overlap rate.

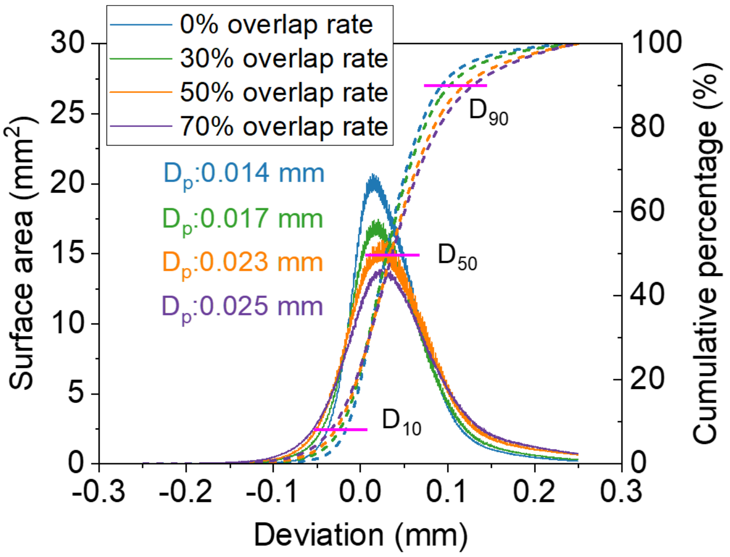

Supplementary Figure 4. Distributions of surface deviations for 3D printed microlattice metamaterials. All surface deviations exhibit an approximately Gaussian

distribution that is not absolutely symmetrical with the peak surface deviation. The cumulative percentages at every D-values stage show that most deviations are positive, signifying the as-built dimensions are larger than those of the as-designed ones. The D-values (D_{10}, D_{50}, and D_{90}) indicate the intercepts for 10%, 50%, and 90% of the cumulative percentage. Source data are provided as a Source Data file.

Supplementary Note 1. Void defects in 3D-printed metamaterial catalysts

For void defects, we conducted micro-CT void defect analysis on the metamaterial structures manufactured by SLM with different overlap rates (Supplementary Figure 5). Firstly, we analyzed the void defects of overall CT-reconstructed metamaterial models. We found that the stainless steel-based metamaterial structures manufactured by SLM have extremely low void defects. For the 15×15×15 mm^3 metamaterial structure with a void defect volume controlled at below 5×10^{-4} mm^3, the peak void volume ranges from 2.5 to 3.5×10^{-5} mm^3. As the overlap rate of the metamaterial increases, the volume of the void defects decreases, which may be due to the tight microlattice structure arrangement, which reduces the accumulation of heat in different regions of the metamaterials, thereby reducing the void defects. 316L stainless steel is a mature raw material for the SLM process. Then, we also used a strut element in the microlattice metamaterials as the region of interest (ROI) for local pore defect analysis (Supplementary Figure 6). The volume of voids is also maintained at a relatively low level, below 10^{-3} mm^3, and as the degree of metamaterial overlap increases, the void defects tend to decrease. The distribution of void defects is mainly located in the central area of the strut element. In addition, suspended molten or semi-molten metal powder particles can also be observed. This is a limitation of the manufacturing accuracy of SLM-printed microlattice metamaterials.

From the results of the CT analysis, it can be seen that the void defects of wood-inspired metamaterials made of 316L stainless steel are controlled at an extremely low level and do not affect the mechanical properties of the metamaterials. Therefore, we consider that the void defect is almost negligible.

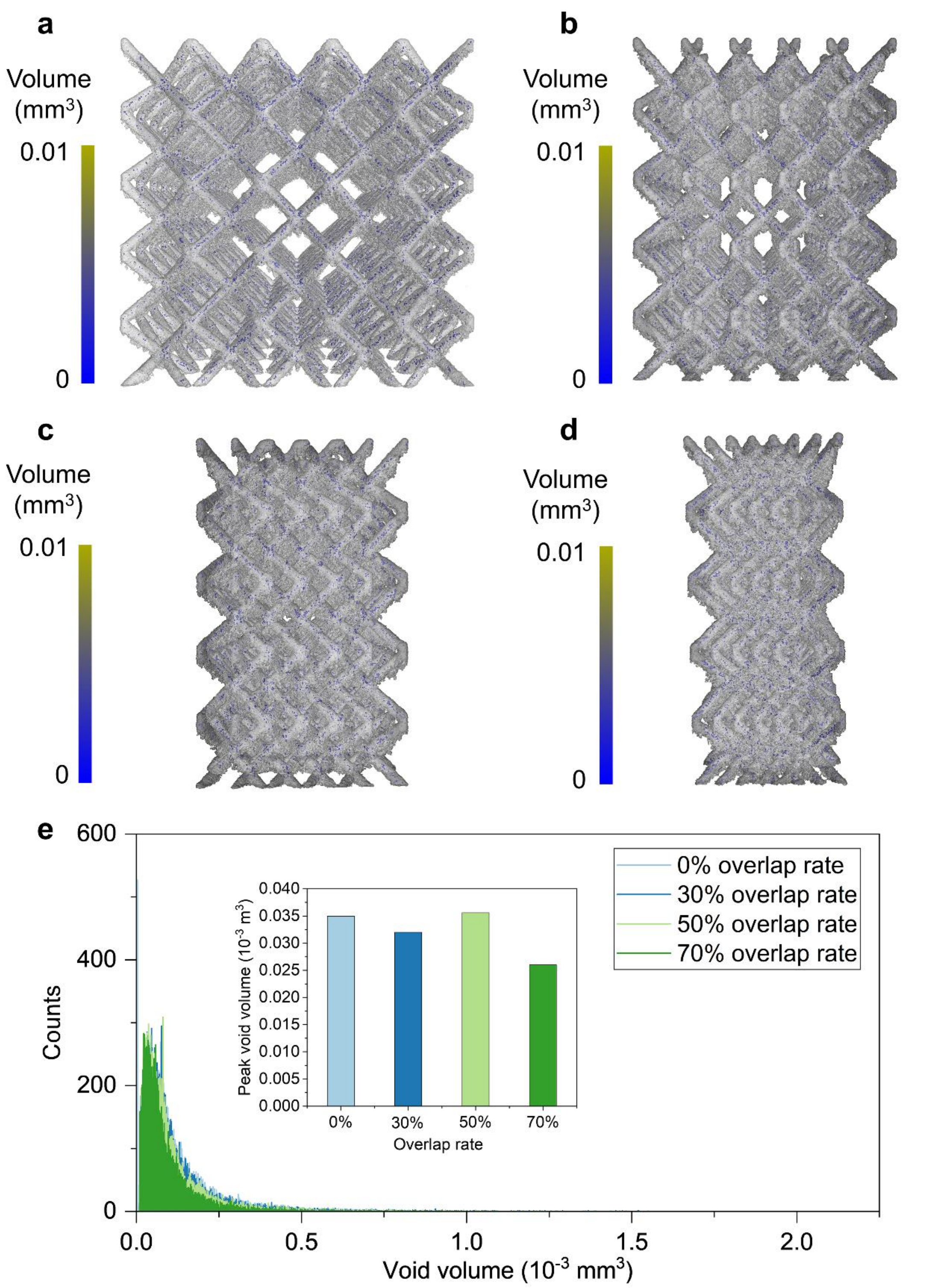

Supplementary Figure 5. The void defect distribution. a, Traditional microlattice with 0% overlap rate and wood-inspired metamaterials with **b,** 30%, **c,** 50%, and **d,** 70% overlap rates. **e,** The comparison of void volume versus counts curves between the traditional microlattice and the wood-inspired metamaterials. Source data are provided as a Source Data file.

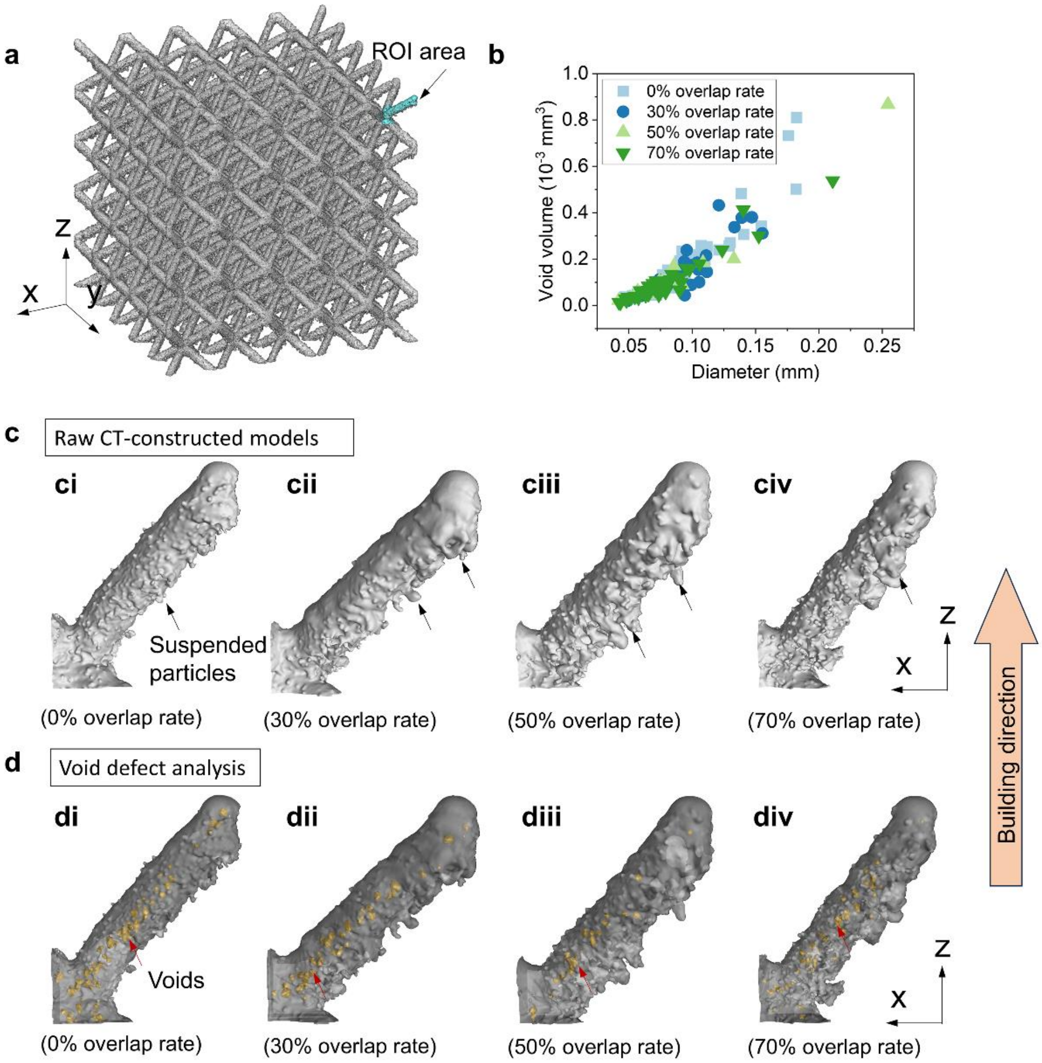

Supplementary Figure 6. The void defect analysis of the region of interest (ROI). **a,** The ROI Schematic model. **b,** The distribution of void defect volume versus their corresponding diameters. **c,** the raw CT-constructed ROI models and **d,** void defect locations for the microlattice (**ci** and **di**) and wood-inspired metamaterials (**cii-civ** and **dii-div**). Source data are provided as a Source Data file.

Supplementary Note 2. Crack defects in 3D-printed metamaterial catalysts

For crack defects, we observed the surface morphology of microlattice metamaterials manufactured by SLM using scanning electron microscopy and did not find any crack defects (Supplementary Figure 7). This is attributed to the excellent additive manufacturing processability of 316L stainless steel substrate.

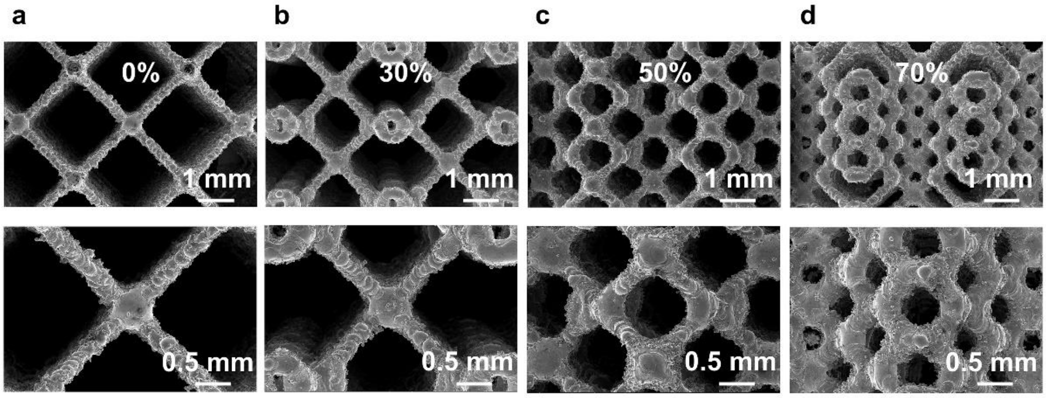

Supplementary Figure 7. The surface morphologies. a, traditional microlattice with 0% overlap rate and wood-inspired metamaterials with **b,** 30%, **c,** 50%, and **d,** 70% overlap rates.

Supplementary Note 3. Manufacturing accuracy of 3D-printed metamaterial catalysts

For manufacturing accuracy, we have compared and analyzed the surface morphology and surface deviation in two-dimensional slices of SLM-built microlattice metamaterials (Supplementary Figure 8). The original micro-CT sections indicate that there is a significant phenomenon of metal powder adhesion on the downward side of the strut elements in the microlattice metamaterials. Two-dimensional surface deviation contours of the microlattices based on the CT data and original models are presented in Supplementary Figure 8e-h. The thin color lines perpendicular to the surfaces of isolated struts signify the deviations while the length and the color type of these lines signify the magnitude of the manufacturing surface deviation. It can be observed that the adhesive particles on the lower surface are the main cause of manufacturing deviation. Supplementary Figure 8i-l shows the statistical surface deviations and their distributions for the traditional microlattices and wood-inspired microlattice metamaterials with different overlap rates. The surface deviations of all microlattices were Gaussian distributed. The peak deviations of the microlattices with 0%, 30%, 50%, and 70% overlap rates were 14 μm, 17 μm, 23 μm, and 25 μm, respectively. The D-values, D10, D50, and D90, i.e., the intercepts for 10%, 50%, and 90% of the

cumulative percentages based on the surface area of the traditional microlattices, were -13 μm, 29 μm, and 93 μm, respectively. The corresponding values for the wood-inspired microlattice metamaterials with 30% overlap rates were -19 μm, 30 μm, and 102 μm. The corresponding values for the wood-inspired microlattice metamaterials with 50% overlap rates were -23 μm, 35 μm, and 119 μm. The corresponding values for the wood-inspired microlattice metamaterials with 30% overlap rates were -27 μm, 37 μm, and 129 μm. It can be found that whether it is the peak deviation or the cumulative deviation, as the overlap rate increases, the absolute value of the deviation shows a gradually increasing trend. In general, wood-inspired microlattice metamaterials with larger overlap rates exhibit a longer thermal history of the interaction between the laser and powder[1]. The alternating heating and cooling processes led to more notable adhesion of the powder to the struts[2]. This adhesion was probably the reason for the higher positive deviation in the wood-inspired metamaterials than in traditional microlattices.

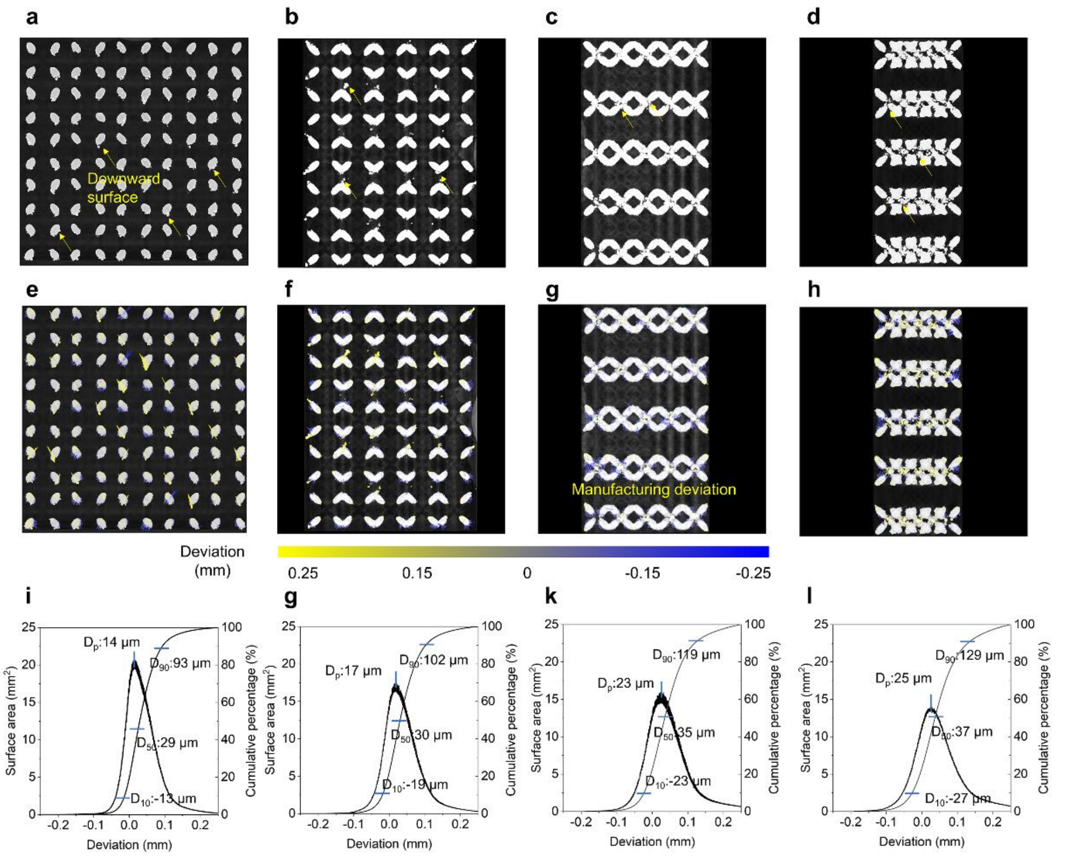

Supplementary Figure 8. The surface morphology and surface deviation in two-dimensional slices. a-d, The CT-reconstructed 2D slicing model, **e-h,** surface deviation

analysis, and **i-l,** the manufacturing deviation data of the **a,** traditional microlattice with 0% overlap rate and wood-inspired metamaterials with **b,** 30%, **c,** 50% and **d,** 70% overlap rates. Source data are provided as a Source Data file.

Supplementary Note 4. Surface area analysis of metamaterial catalysts

However, when using SLM-manufactured microlattice metamaterials for catalytic applications, the manufacturing accuracy would increase the available surface area of metamaterial catalysts (Supplementary Figure 9), thereby improving catalytic efficiency. It can be observed that as the overlap rate increases, the improvement rate of surface area of SLM-manufactured metamaterials also gradually increases (Supplementary Figure 9). Correspondingly, the normalized degradation kinetic constant increases with the increase in overlap rate (Fig. 4a).

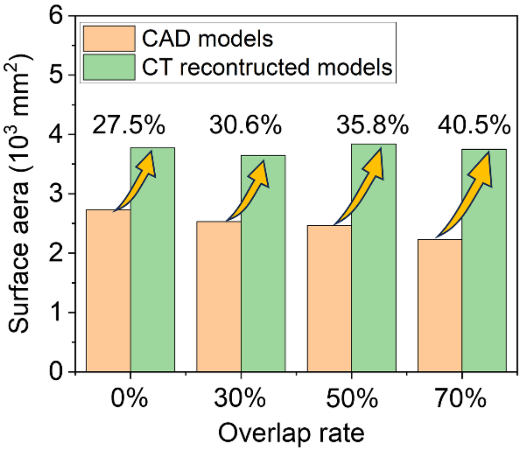

Supplementary Figure 9. Surface area comparison between CAD models and CT-reconstructed models for the microlattices and wood-inspired metamaterials with different overlap rates. Source data are provided as a Source Data file.

Supplementary Note 5. Von Mises stress distribution of metamaterial catalysts

We have performed mechanical simulation compression on the CT-reconstructed models of the raw SLM-manufactured samples, and compared them with the CAD structural model, with one end fixed and the other end subjected to a pressure of 1 Pa (Fig. S10a). We found that the stress level of SLM-manufactured structures is greater than that of CAD model structures (Fig. S10b), which could be attributed to the local

surface deviations and void defects. For both CAD models and CT-reconstructed models, the increasing overlap rate of microlattices gradually increases their structural stiffness, resulting in an increase in the maximum stress inside the structure. Besides, the slight manufacturing deviations and negligible pore defects do not significantly alter the stress distribution, but only marginally increase the degree of stress concentration (Fig. S10c-f).

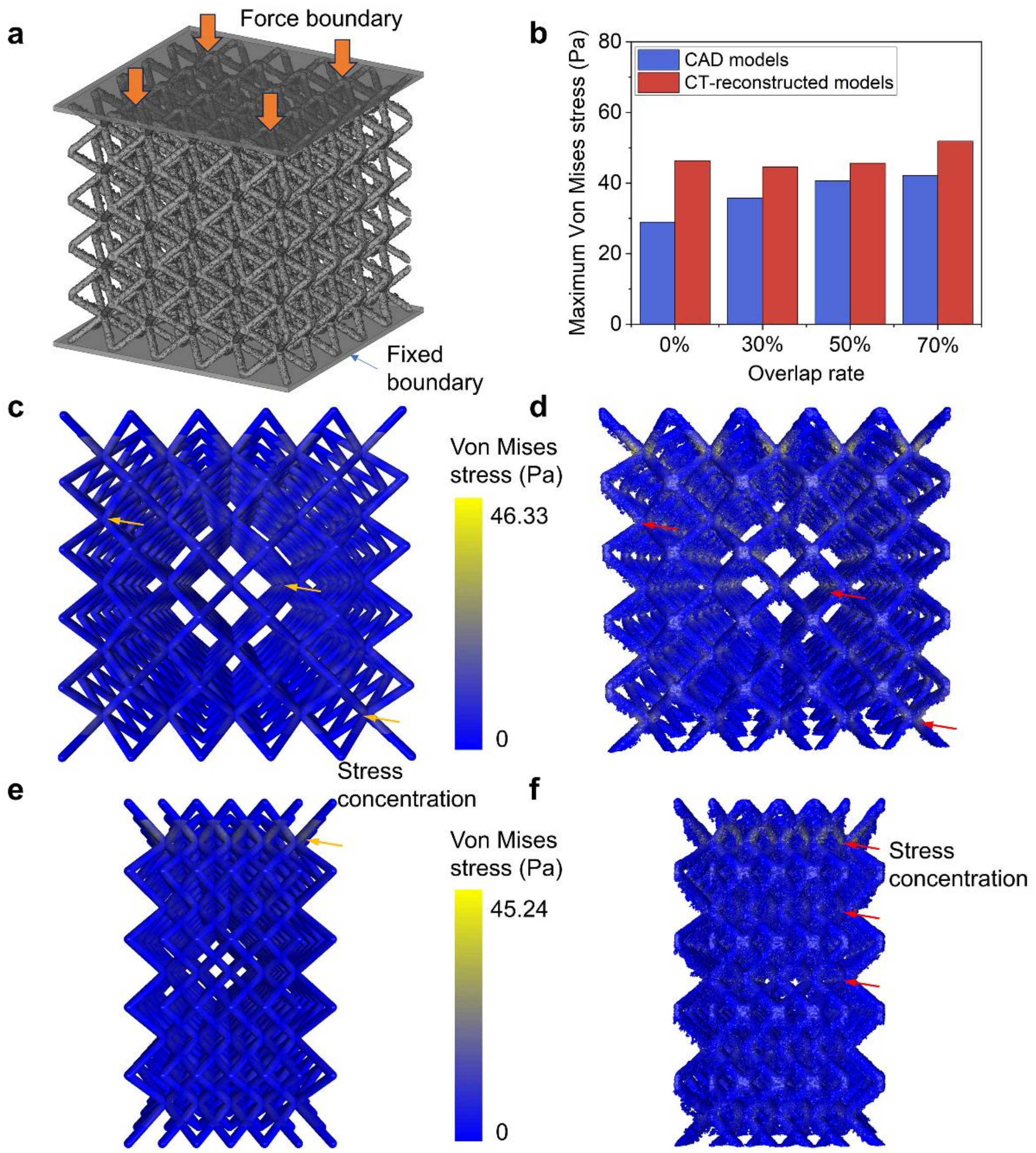

Supplementary Figure 10. Von Mises stress distribution comparison between CAD models and CT-reconstructed models for traditional microlattices and wood-inspired metamaterials. a, Boundary condition. **b,** Maximum Von mises stress

comparison of wood-inspired metamaterials under different overlap rates and traditional microlattices. Stress distribution of **c,** the CAD model and **d,** the CT-reconstructed model of the traditional microlattice with 0% overlap rate. Stress distribution of **e,** the CAD model and **f,** the CT-reconstructed model of the wood-inspired metamaterial with 50% overlap rate. Source data are provided as a Source Data file.

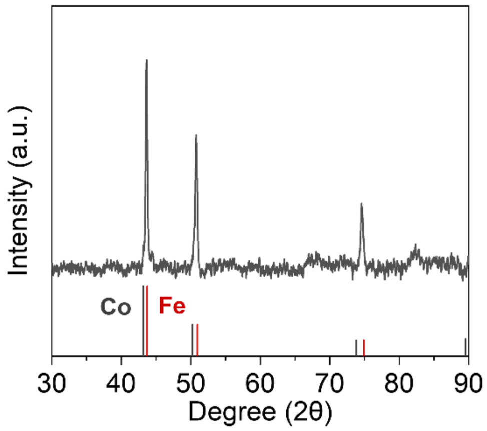

Supplementary Figure 11. X-ray diffraction curve of Co/Fe wood-inspired metamaterial catalyst, indicating the successful synthesis of the material. Source data are provided as a Source Data file.

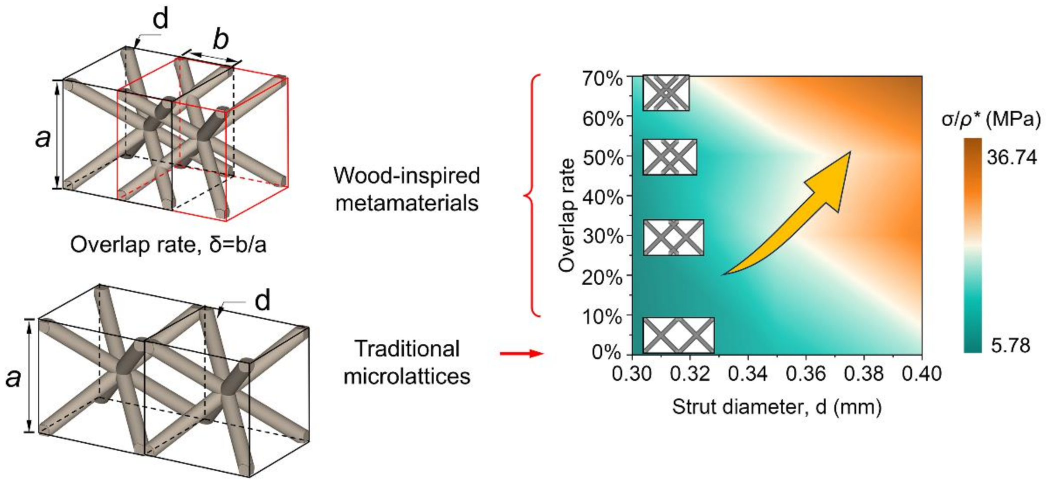

Supplementary Figure 12. Contour map of normalized strength under different overlap rates and strut diameters for the wood-inspired metamaterial catalyst. Source data are provided as a Source Data file.

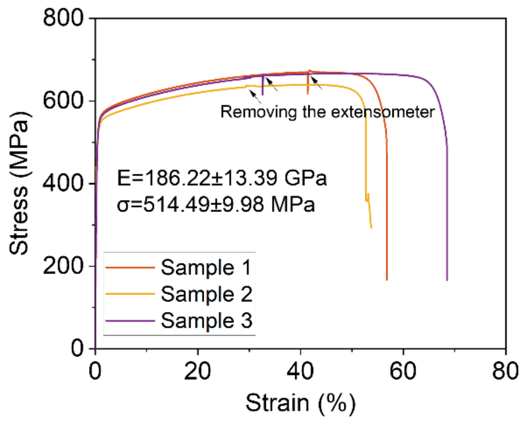

Supplementary Figure 13. The stress-strain curves of 316L stainless steel. Source data are provided as a Source Data file.

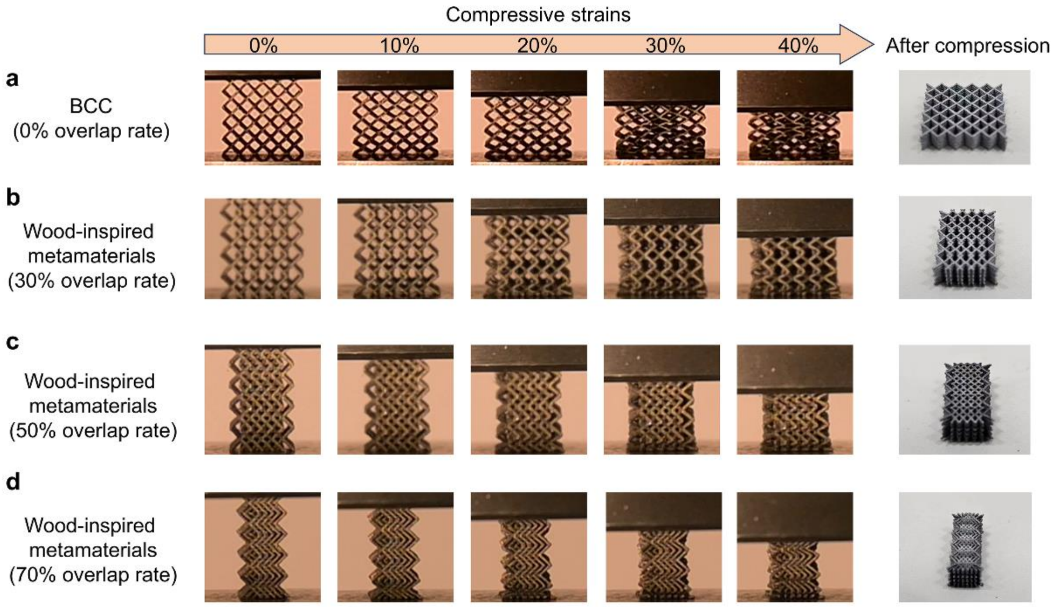

Supplementary Figure 14. Compression deformation behavior of microlattice metamaterials with different overlap rates. **a,** 0%, **b,** 30%, **c,** 50%, and **d,** 70% overlap rates.

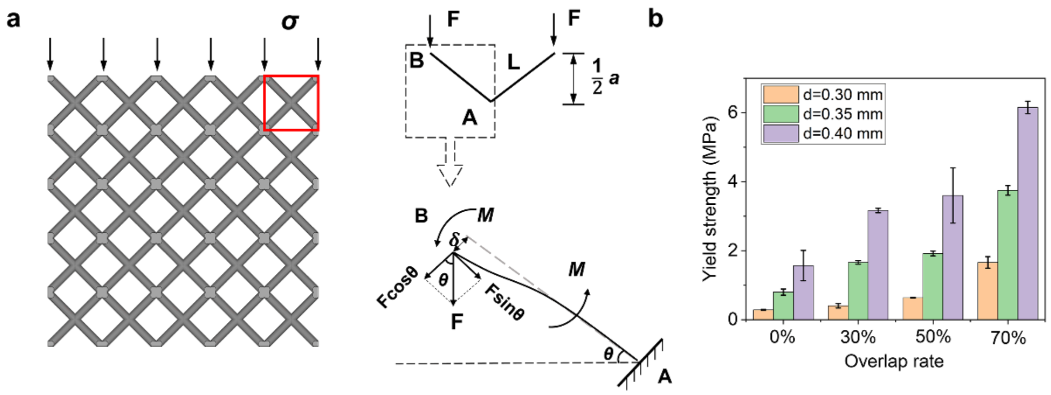

Supplementary Figure 15. Schematic of BCC-based microlattice metamaterials loaded in longitudinal direction. a, boundary conditions, the force applied to a quarter model of an arbitrary unit cell and free body diagram of an inclined strut. **b,** The yield strength comparison of microlattice metamaterials with different overlap rates. Error bars represent standard deviation (n = 3). Source data are provided as a Source Data file.

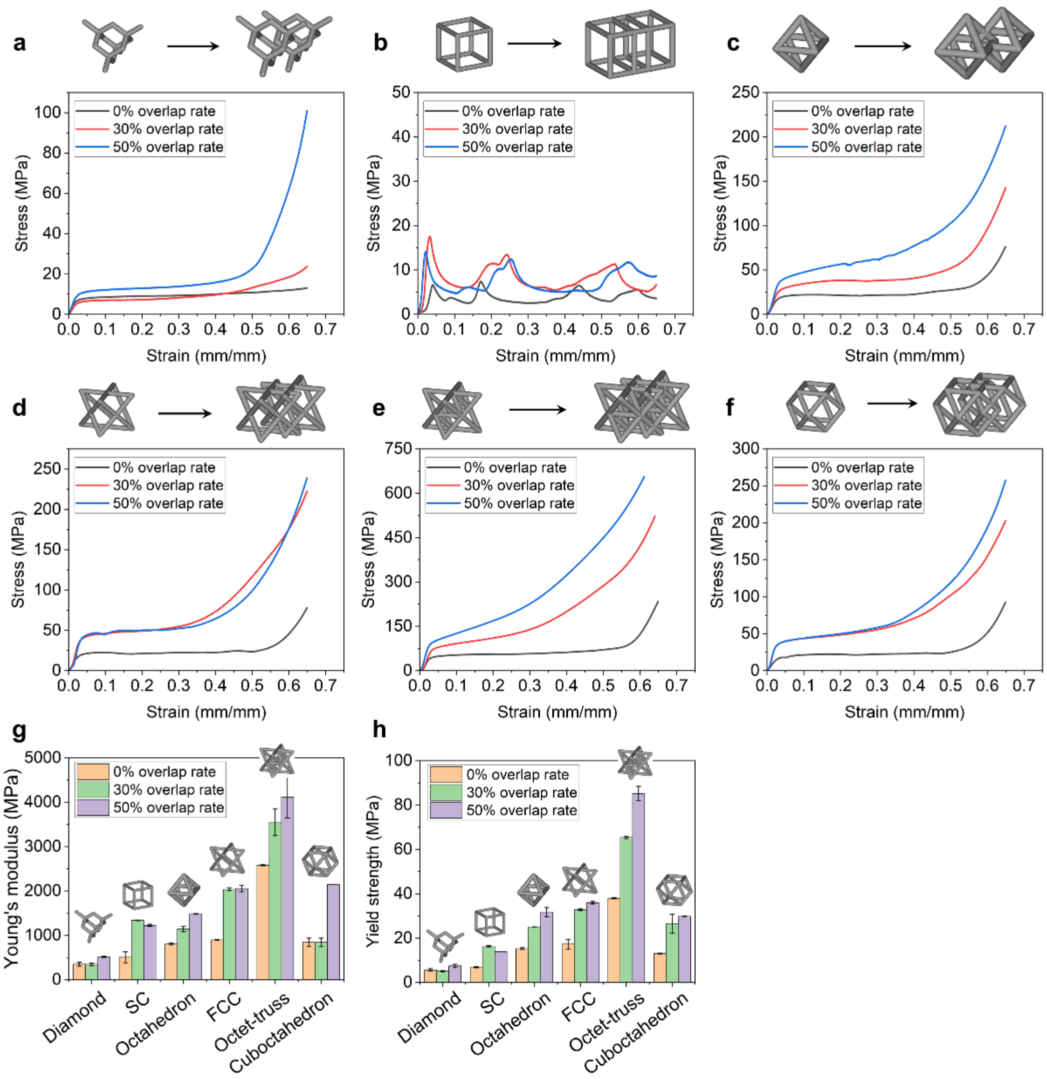

Supplementary Figure 16. The mechanical responses of different traditional topological microlattices and their corresponding wood-inspired metamaterials. a-f, stress-strain curve results and **g,** Young's modulus and **h,** yield strength. The traditional microlattices are **a,** diamond, **b,** simple cubic (SC), **c,** octahedron, **d,** face-center cubic (FCC), **e,** octet-truss, and **f,** cuboctahedron, respectively. All error bars represent standard deviation (n = 3). Source data are provided as a Source Data file.

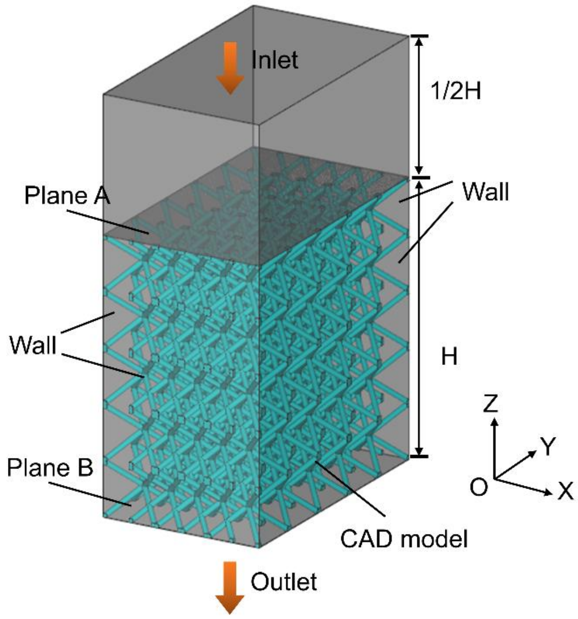

Supplementary Figure 17. Computational fluid dynamics models and boundary conditions. Considering the influence of the boundary effect, the above void area was added to the CAD model, in which the height of the void area was half of the microlattices. The velocity of the inlet-flow side was 0.001 m s^{-1} while the outlet pressure of the opposite-flow side was set as zero[3].

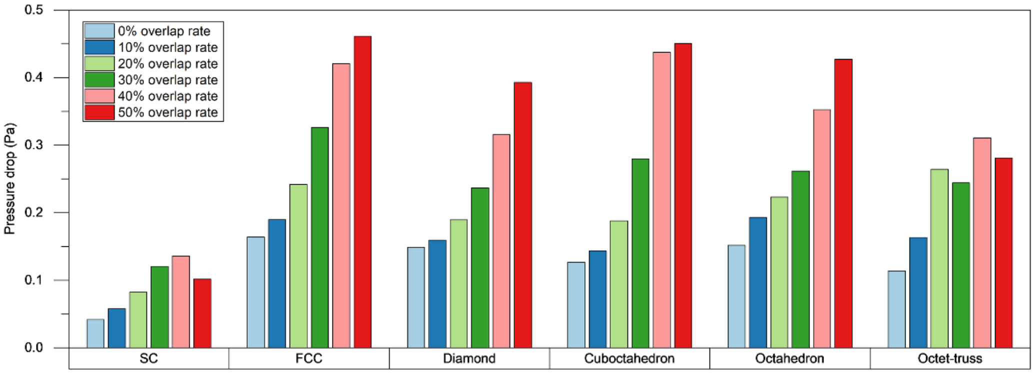

Supplementary Figure 18. Comparison of the apparent pressure drop of microlattice metamaterials under different overlap rates and structural types as a function of relative density. The results show that the wood-inspired metamaterial has a higher pressure drop than the traditional periodic microlattices. Source data are provided as a Source Data file.

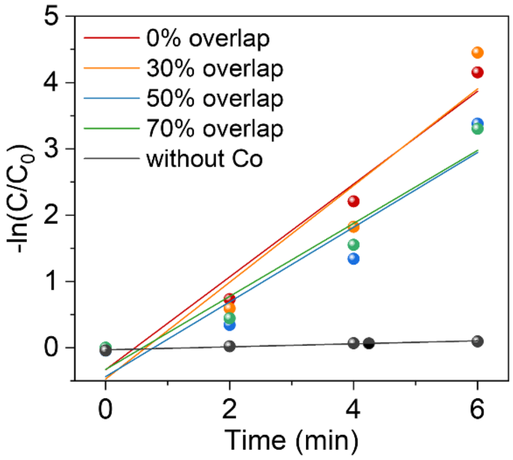

Supplementary Figure 19. Reaction kinetic curves of Co/SS metamaterial catalysts with different overlap rates after fitting. For comparison, the 70%-overlap metamaterial catalyst without Co coating was investigated, and it exhibited an SMX degradation rate of only 16% after 15 min treatment. This confirms that the Co decoration played a crucial role in the PMS activation performance. Source data are provided as a Source Data file.

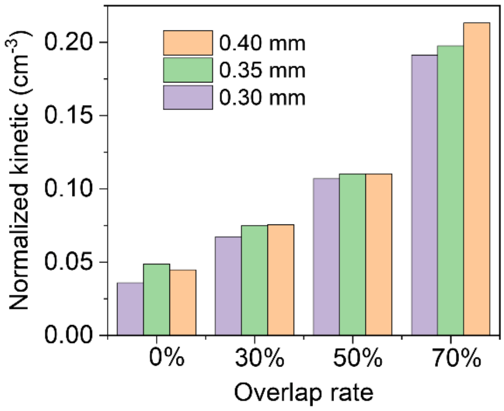

Supplementary Figure 20. The kinetic constants of the wood-inspired metamaterial catalyst activation PMS with different strut diameters. Source data are provided as a Source Data file.

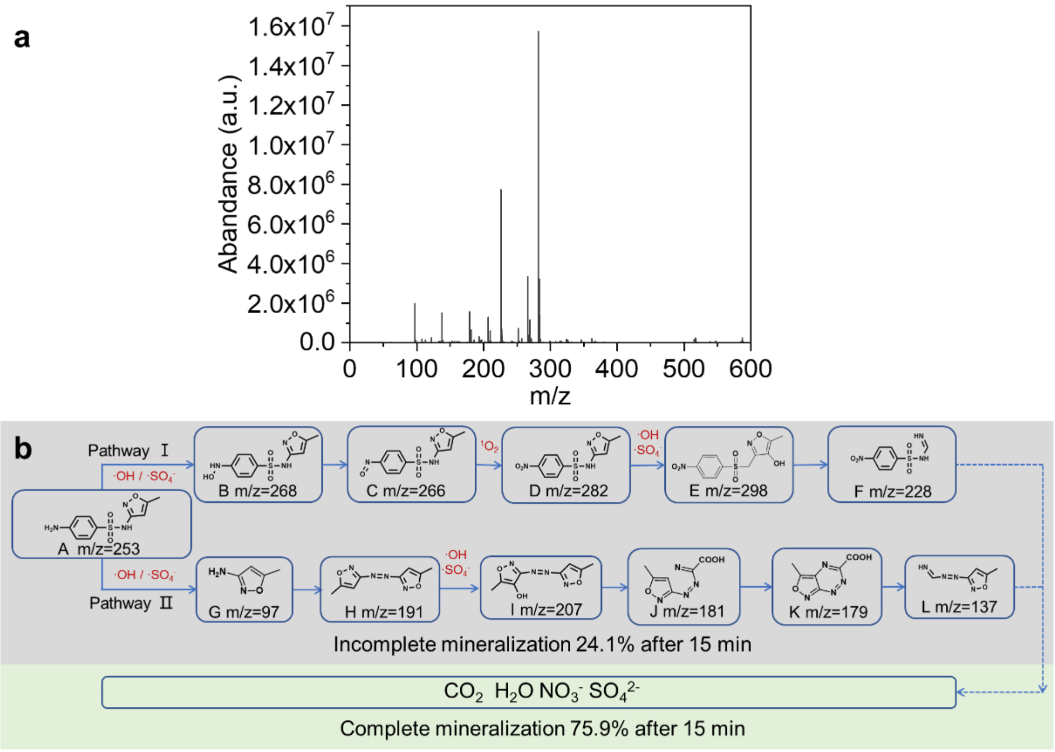

Supplementary Figure 21. Reaction intermediates detected by HPLC-MS and inference of SMX degradation pathway. a, The mass spectrometry of the possible intermediate products. **b,** SMX degradation pathway inferred from HPLC-MS results. Source data are provided as a Source Data file.

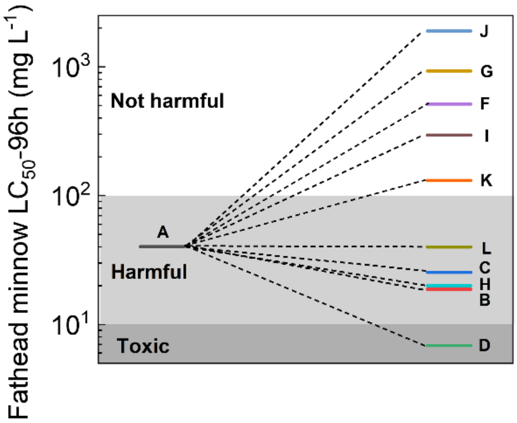

Supplementary Figure 22. Fathead minnow LC$_{50}$-96 h of SMX and degradation intermediates.

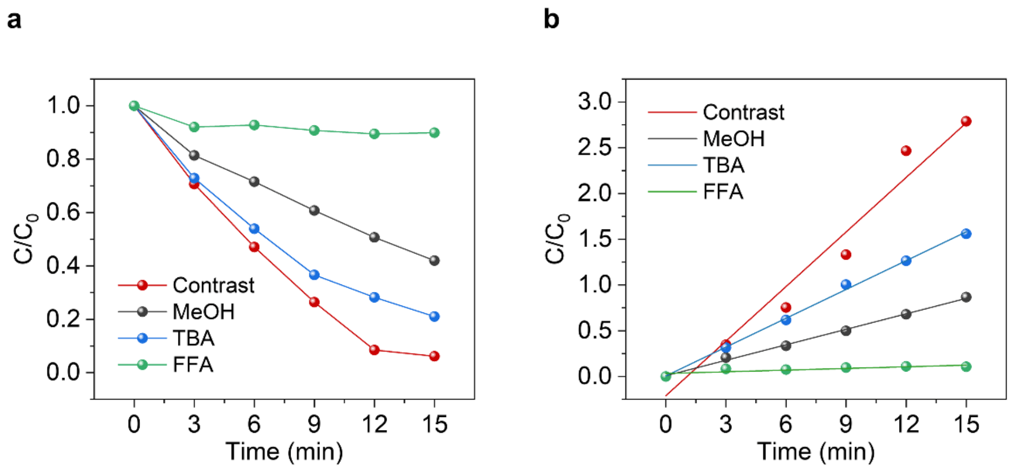

Supplementary Figure 23. a, The degradation results and **b,** fitting kinetic curve of the degradation system after the addition of different quenchers. Source data are provided as a Source Data file.

Supplementary Note 6. Corrosion resistance analysis of metamaterial catalysts

The corrosion resistance of Co/SS metamaterial catalysts is the basement for long-term use. To investigate the effect of cobalt coating on the corrosion resistance of SS-based metamaterials in the operating environment, the polarization current curve of Co/SS metamaterial catalysts and SS-based metamaterials in 0.2 g L^{-1} PMS solution and DI water was tested, as shown in Supplementary Figure 24a, and the corrosion potential and corrosion current density were recorded at Supplementary Figure 24b-c. It can be seen that in 0.2 g L^{-1} PMS solution, the corrosion potential E_c of Co/SS metamaterial catalysts and SS-based metamaterials are -0.58 V and -0.09 V, respectively, where corrosion current I_c of Co/SS metamaterial catalysts and SS-based metamaterials are 0.47 μA cm^{-2} and 0.14 μA cm^{-2}). While in DI water, the corrosion potential E_c of Co/SS metamaterial catalysts and SS-based metamaterials are -0.44 V and -0.13 V, respectively, where corrosion current I_c of Co/SS metamaterial catalysts and SS-based metamaterials are 0.15 μA cm^{-2} and 0.035 μA cm^{-2}). This is the response for Co as a reactive substance is not as resistant to corrosion of PMS as Fe.

According to the standard electrode potential $E^{\ominus}$ of Fe/Fe^{2+} is -0.44 V and $E^{\ominus}$ of Co/Co^{2+} is -0.28 V, which means that galvanic corrosion occurs when Co/SS contact, and electrons are transferred from Fe to Co. This result hurts the corrosion resistance of

SS-based metamaterials as shown in the above corrosion data. However, electron injection into Co is conducive to the circulation of Co species in the catalytic process. This is an important reason for the good catalytic performance of Co/SS metamaterial catalysts. As shown in Supplementary Figure 24d, the process of PMS activation will increase the valence state of Co species, and the high-valence state of Co will inhibit the activation process of PMS to obtain electrons. The galvanic current between Co/SS can inject Fe electrons into Co species to promote the reduction of Co, which accelerates the cycle process of Co species. It is beneficial to improve the activity and prolong the service life. It is worth noting that the Fe in metamaterial catalysts is bulky and large, the galvanizing corrosion between Fe metamaterials with surface Co will not cause serious damage to the metamaterial catalysts structure.

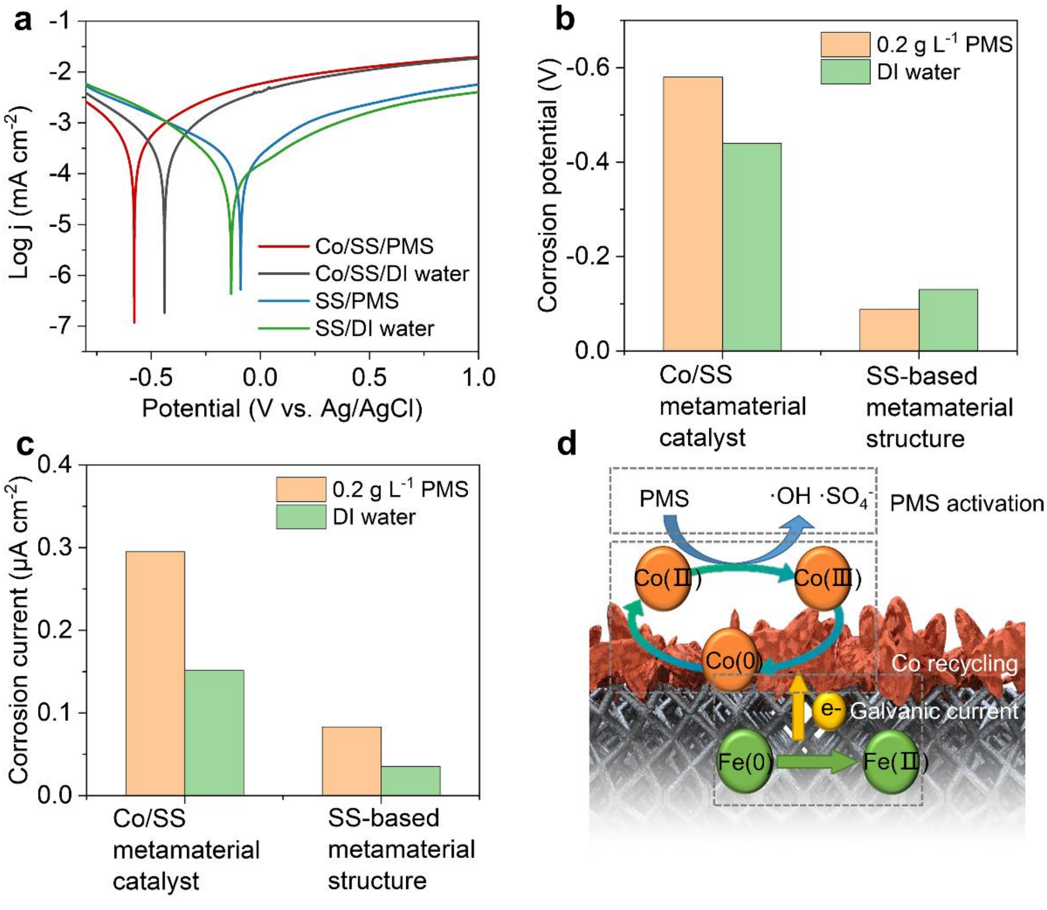

Supplementary Figure 24. Corrosion resistance analysis of metamaterial catalysts. **a,** Polarization current curves of Co/SS and SS metamaterial in 0.2 g L^{-1} PMS solution and DI water, respectively. **b,** Corrosion potential **c,** corrosion current comparison of Co/SS and SS metamaterial in 0.2 g L^{-1} PMS solution and DI water, respectively. **d,**

Schematic diagram of a galvanic current promoting the circulation of Co species. Source data are provided as a Source Data file.

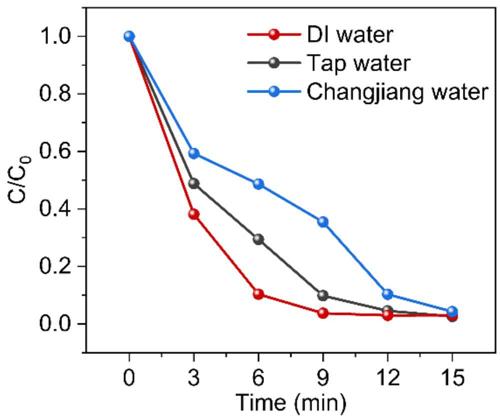

Supplementary Figure 25. The actual water samples as the solvent to test the activation degradation of SMX by PMS. Source data are provided as a Source Data file.

Supplementary Note 7. Comparison between wood and wood-inspired metamaterials

Wood and wood-inspired metamaterials have completely different materials and different pore scales. The pore size of Douglas fir (0.1 mm diameter) is about one-seventh that of microlattice metamaterials with 70% overlap rates (0.75 mm diameter) Supplementary Figure 26a). Wood with micrometer-scale pores has good mechanical properties (Supplementary Figure 26b-c), but also significantly reduces its transport performance (Supplementary Figure 26d). On the contrary, wood-inspired metamaterials with millimeter-scale pores have moderate modulus/strength and excellent transport performances.

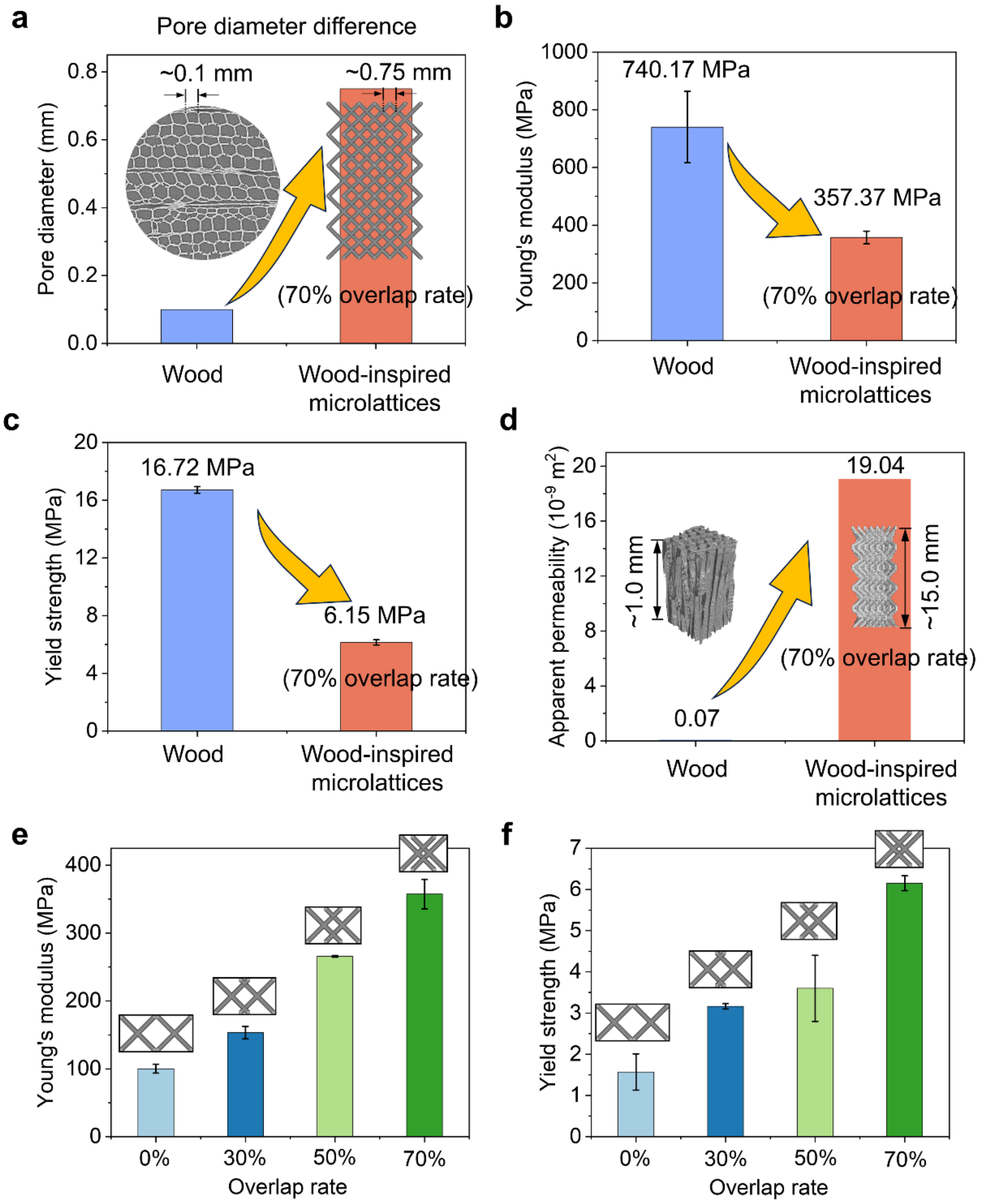

Supplementary Figure 26. Comparison between the wood of Douglas fir and wood-inspired metamaterials. a, pore size, **b,** Young's modulus, **c,** yield strength, and **d,** apparent permeability. Improvement in **e,** Young's modulus and **f,** yield strength through wood-inspired overlapping microlattice design strategy. All error bars represent standard deviation (n = 3). Source data are provided as a Source Data file.

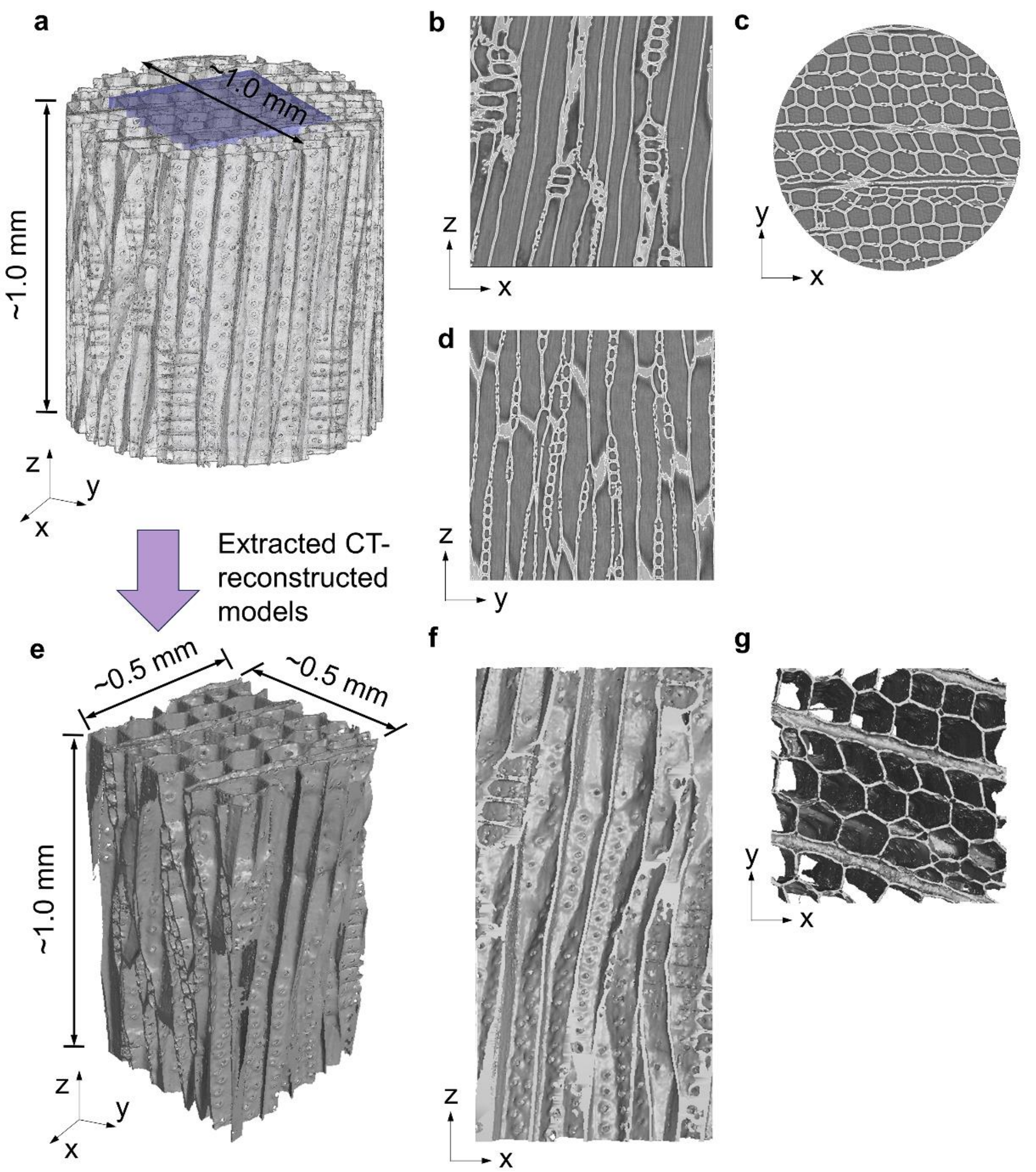

Supplementary Figure 27. Microstructures of wood. a-d) CT-reconstructed wood microstructures: **a**) The CT-reconstructed wood models. **b**) The x-z plane view. **c**) The x-y plane view. **d**) The x-y plane view. **e-g**) The CT-reconstructed region of interest (ROI) models: **e**) the ROI was extracted from the CT-reconstructed wood models. **f**) The x-z plane view. **g**) The x-z plane view. The wood is scanned by the X-ray microscopes (ZEISS Xradia Versa 610). The applied voltage in the micro-CT measurements was 60 kV. These reconstructed models consist of isotropic voxels with 0.5 μm and take a scanning period of 150 min. The Air filter was used for scanning the wood sample. These scanning data were further analyzed and visualized using the commercially available image analysis software VG Studio Max 3.4 (Volume Graphics

GmbH, Heidelberg, Germany).

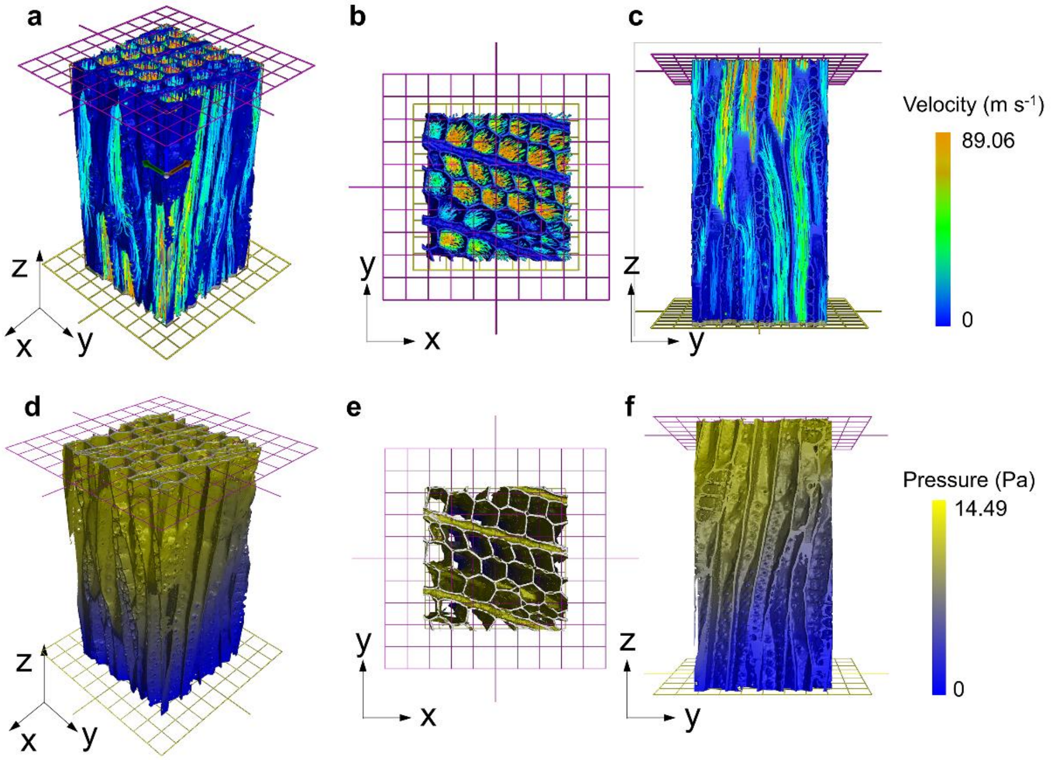

Supplementary Figure 28. Transport performance of wood. a-c, The velocity distribution under different views. **d-f,** The pressure distribution under different views. The upper surface is the inlet boundary and the downward surface is the outlet boundary. Aside from the surfaces of the inlet and outlet, the other walls are set as wall boundaries without slip conditions. The velocity of the inlet-flow side is 0.001 m s^{-1} while the outlet pressure of the opposite-flow side is set as zero. The physical properties of fluid with a density of 1000 kg m^{-3} and a viscosity of 1.01×10^{-3} Pa s were assigned to the liquid domain.

Supplementary Table 1. Comparison of TOC removal rates and the reaction time for some catalysts.

No.	Catalyst	Pollutant	Reaction time	TOC removal	Ref.
1	Co/SS metamaterial catalysts	SMX	15 min	75.9%	This work
2	SA-Cu/rGO	SMX	54 min	60%	[4]
3	$CuO@FeO_x@Fe^0$	SMX	10 min	50.5%	[5]
4	EC/Fe（III）	SMX	81 min	63.12%	[6]
5	$Co/CoO/Co_9S_8@NSOC$	SMX	10 min	32.80%	[7]
6	$FeSe_2@MoO_3$-8/PVDF	2,4-D	8 h	70%	[8]
7	ZVI	PhOH	20 min	22%	[9]

Supplementary Table 2. Summarizes the stability working time for some catalysts.

No.	Catalyst	Pollutant	Pollutant concentration (mg L^{-1})	Oxidant concentration	Reaction time	Ref.
1	Co/SS metamaterial catalysts	SMX	10	PMS 0.2 g L^{-1}	7 d	This work
2	zeolite@ZIF-67 composites	TC	50	PMS 0.5 g L^{-1}	300 min	[10]
2	3DP MG/30Cu	RHB	20	H_2O_2 2 mM	over 100 cycles	[22]
3	ce-MoS2	BPA	2	PMS 0.05 g L^{-1}	360 min	[23]
4	CoS@FeS-1	SMX	10	PMS 1 mM	15 h	[24]
5	Co-TPML	BPA	50 μM	PMS 20 mM	60 min	[25]
6	BA/MoS_2@CSH	DC	40	PMS 0.333 g L^{-1}	3500 min	[26]

Supplementary Table 3. General characteristics comparison of the common materials used for water purification.

Types	Material	Manufacturing	Structural designability	Mechanical robustness	Controllability	Functionality	Ref
Traditional microlattices	Metallic glass	3D printing	√ √ √ √	√ √ √ √ √	√ √ √ √	√	11
	TiCu alloy	3D Printing and chemical dealloying	√ √ √ √	√ √ √ √	√ √ √ √	√ √	12
	Nylon	3D printing	√ √ √ √	√ √ √	√ √ √ √	√	13
	316L	3D printing	√ √ √ √	√ √ √ √ √	√ √ √ √	√	14
	Pure copper	3D printing	√ √ √ √	√ √ √	√ √ √ √	√ √ √	15
Synthetic catalyst/porous carbon	ce-MoS_2	Lamellar membrane	√ √	√	√ √ √	√ √ √ √	16
	DSLB-800	Thermal treatment	√ √	√ √	√ √	√ √ √ √	17
	CoS@FeS-1	Hydrothermal method	√	√	√ √	√ √ √	18
	Co_3O_4/C@SiO_2	A modified NIPS method	√	√	√ √	√ √ √ √	19
	Co-TPML	Chemical synthesis	√	√	√	√ √ √	20
	CuO@CHFMs	A phase-inversion process	√	√	√ √	√ √ √ √	21
This work	316L and Co	3D printing and ECD process	√ √ √ √ √	√ √ √ √ √	√ √ √ √ √	√ √ √ √ √	

DSLB: Distilled spirits lees derived biochar

NIPS: Nonsolvent-induced phase separation

Co-TPML: Tetrapyridomacrocyclic ligand-bound cobalt

CuO@CHFMs: CuO-coated ceramic hollow fiber membranes

ECD: Electrochemical deposition

Supplementary References

1 Yang, L. *et al.* An investigation into the effect of gradients on the manufacturing fidelity of triply periodic minimal surface structures with graded density fabricated by selective laser melting. *Journal of Materials Processing Technology* **275**, 116367, doi:10.1016/j.jmatprotec.2019.116367 (2020).

2 Al-Ketan, O., Rowshan, R. & Abu Al-Rub, R. K. Topology-mechanical property relationship of 3D printed strut, skeletal, and sheet based periodic metallic cellular materials. *Additive Manufacturing* **19**, 167-183, doi:10.1016/j.addma.2017.12.006 (2018).

3 Zhang, L., Song, B., Yang, L. & Shi, Y. Tailored mechanical response and mass transport characteristic of selective laser melted porous metallic biomaterials for bone scaffolds. *Acta Biomaterialia* **112**, 298–315, doi:10.1016/j.actbio.2020.05.038 (2020).

4 He, Y., Zhang, J., Zhou, H., Yao, G. & Lai, B. Synergistic multiple active species for the degradation of sulfamethoxazole by peroxymonosulfate in the presence of CuO@FeOx@Fe0. *Chemical Engineering Journal* **380**, doi:10.1016/j.cej.2019.122568 (2020).

5 Chen, F. *et al.* Efficient degradation and mineralization of antibiotics via heterogeneous activation of peroxymonosulfate by using graphene supported single-atom Cu catalyst. *Chemical Engineering Journal* **394**, doi:10.1016/j.cej.2020.124904 (2020).

6 Shizong, W. & Jianlong, W. Synergistic effect of PMS activation by Fe0@Fe3O4 anchored on N, S, O co-doped carbon composite for degradation of sulfamethoxazole. *Chemical Engineering Journal* **427**, 131960 (2022).

7 Jiang, Y. *et al.* Superhydrophilic N,S,O-doped Co/CoO/Co9S8@carbon derived from metal-organic framework for activating peroxymonosulfate to degrade sulfamethoxazole: Performance, mechanism insight and large-scale application. *Chemical Engineering Journal* **446**, doi:10.1016/j.cej.2022.137361 (2022).

8 Wang, F. *et al.* The Pivotal Role of Selenium Vacancies in Defective FeSe2@MoO3 for Efficient Peroxymonosulfate Activation: Experimental and DFT Calculation. *ACS ES&T Engineering*, doi:10.1021/acsestengg.3c00195 (2023).

9 Zhang, Y. J. *et al.* Simultaneous nanocatalytic surface activation of pollutants and oxidants for highly efficient water decontamination. *Nat Commun* **13**, 3005, doi:10.1038/s41467-022-30560-9 (2022).

10 Chen, D. *et al.* Stable metal–organic framework fixing within zeolite beads for effectively static and continuous flow degradation of tetracycline by peroxymonosulfate activation. *Chemical Engineering Journal* **435**, doi:10.1016/j.cej.2022.134916 (2022).

11 Yang, C. *et al.* Three-Dimensional Hierarchical Porous Structures of Metallic Glass/Copper Composite Catalysts by 3D Printing for Efficient Wastewater Treatments. *ACS Appl Mater Interfaces* **13**, 7227-7237, doi:10.1021/acsami.0c20832 (2021).

12 Cai, C. *et al.* 3D Printing and Chemical Dealloying of a Hierarchically Micro- and Nanoporous Catalyst for Wastewater Purification. *ACS Appl Mater Interfaces* **13**, 48709-48719, doi:10.1021/acsami.1c14076 (2021).

13 Wang, Y., Li, L., Hofmann, D., Andrade, J. E. & Daraio, C. Structured fabrics with tunable mechanical properties. *Nature* **596**, 238-243, doi:10.1038/s41586-021-03698-7 (2021).

14 Pham, M. S., Liu, C., Todd, I. & Lertthanasarn, J. Damage-tolerant architected materials inspired by crystal microstructure. *Nature* **565**, 305-311, doi:10.1038/s41586-018-0850-3

(2019).
15 Guo, S. *et al.* 3D printed hierarchically porous zero-valent copper for efficient pollutant degradation through peroxymonosulfate activation. *Separation and Purification Technology* **305**, doi:10.1016/j.seppur.2022.122437 (2023).
16 Chen, Y., Zhang, G., Liu, H. & Qu, J. Confining Free Radicals in Close Vicinity to Contaminants Enables Ultrafast Fenton-like Processes in the Interspacing of MoS(2) Membranes. *Angew Chem Int Ed Engl* **58**, 8134-8138, doi:10.1002/anie.201903531 (2019).
17 Wang, Y. *et al.* Sulfamethoxazole degradation by regulating active sites on distilled spirits lees-derived biochar in a continuous flow fixed bed peroxymonosulfate reactor. *Applied Catalysis B: Environmental* **310**, doi:10.1016/j.apcatb.2022.121342 (2022).
18 Wu, L. *et al.* The synergy of sulfur vacancies and heterostructure on CoS@FeS nanosheets for boosting the peroxymonosulfate activation. *Chemical Engineering Journal* **446**, doi:10.1016/j.cej.2022.136759 (2022).
19 Xie, J. *et al.* Sequential Ultrafiltration-Catalysis Membrane for Excellent Removal of Multiple Pollutants in Water. *Environ Sci Technol* **55**, 2652-2661, doi:10.1021/acs.est.0c07418 (2021).
20 Chu, C. *et al.* Cobalt Single Atoms on Tetrapyridomacrocyclic Support for Efficient Peroxymonosulfate Activation. *Environ Sci Technol* **55**, 1242-1250, doi:10.1021/acs.est.0c06086 (2021).
21 Wang, S. *et al.* Development of CuO coated ceramic hollow fiber membrane for peroxymonosulfate activation: a highly efficient singlet oxygen-dominated oxidation process for bisphenol a degradation. *Applied Catalysis B: Environmental* **256**, doi:10.1016/j.apcatb.2019.117783 (2019).
22 Yang, C. *et al.* Three-Dimensional Hierarchical Porous Structures of Metallic Glass/Copper Composite Catalysts by 3D Printing for Efficient Wastewater Treatments. *ACS Appl Mater Inter* **13**, 7227-7237, doi:10.1021/acsami.0c20832 (2021).
23 Chen, Y., Zhang, G., Liu, H. & Qu, J. Confining Free Radicals in Close Vicinity to Contaminants Enables Ultrafast Fenton-like Processes in the Interspacing of MoS2 Membranes. *Ange Chem Inter Ed* **58**, 8134-8138, doi:https://doi.org/10.1002/anie.201903531 (2019).
24 Wu, L. *et al.* The synergy of sulfur vacancies and heterostructure on CoS@FeS nanosheets for boosting the peroxymonosulfate activation. *Chem Eng J* **446**, 136759, doi:https://doi.org/10.1016/j.cej.2022.136759 (2022).
25 Chu, C. *et al.* Cobalt Single Atoms on Tetrapyridomacrocyclic Support for Efficient Peroxymonosulfate Activation. *Environ Sci Technol* **55**, 1242-1250, doi:10.1021/acs.est.0c06086 (2021).
26 Zhang, Y.-J. *et al.* Simultaneous nanocatalytic surface activation of pollutants and oxidants for highly efficient water decontamination. *Nat Commun* **13**, 3005, doi:10.1038/s41467-022-30560-9 (2022).
